# Delayed Diagnosis: Tuberculous Arthritis of Right Knee Joint in a Patient with Rheumatoid Arthritis

**DOI:** 10.1155/2021/7751509

**Published:** 2021-12-26

**Authors:** H. Senarathna, K. Deshapriya

**Affiliations:** Department of Rheumatology and Rehabilitation, Teaching Hospital, Karapitiya, Sri Lanka

## Abstract

**Background:**

Though skeletal tuberculosis (TB) accounts about 3% of all TB cases, it occupies 10–35% of extrapulmonary TB cases. Common osteoarticular sites involved include the spine (40%), hip (25%), and knee (8%). Co-occurrence of rheumatoid arthritis (RA) and tuberculous arthritis involving peripheral joint is rarely reported in the literature. *Case Presentation*. We present a case of 42-year-old Sri Lankan-Sinhalese male with right knee joint pain and swelling for one-year duration. This patient had a history of long-standing RA with interstitial lung disease for which he was on multiple immunosuppressive medications including methotrexate, sulfasalazine, leflunomide, mycophenolate mofetil, and prednisolone. His knee joint aspiration fluid was positive for both acid fast bacilli (AFB) and polymerase chain reaction for TB (TB-PCR). He was started on anti-tuberculous chemotherapy.

**Conclusion:**

TB should be considered as an important differential diagnosis for chronic mono-arthritis of knee joint with a high degree of suspicion, particularly where TB is endemic.

## 1. Introduction

Tuberculosis (TB) is a major health problem worldwide, particularly in the developing countries. However, the primary organ affected is mostly lung, and skeletal TB accounts 10–35% of extrapulmonary TB (skeletal TB reported up to 3% of all TB cases) [1]. Skeletal TB follows lympho-hematogenous spread from a primary organ in the form of spondylitis (Pott's disease), tuberculous arthritis, osteomyelitis, and Poncet's disease. Common osteoarticular sites involved are the spine (40%), followed by hip (25%) and knee (8%) [2]. But surprisingly, half of the patients with skeletal TB do not show any clinical or radiological evidence of lung involvement [3].

Clinical manifestations of tuberculous arthritis include pain, swelling, and reduced range of motion of the affected joint, but other inflammatory signs such as erythema and warmth may be absent. Only one-third of patients with skeletal TB reported fever and weight loss [4]. Inflammatory markers are often elevated. Combination of periarticular osteopenia, subchondral erosions, and joint space narrowing in plain radiograph of the affected joint (Phemister triad) is not specific but suggestive of tuberculous arthritis. Magnetic resonance imaging (MRI) is increasingly used, but it also gives non-specific findings. Definite diagnosis requires positive culture from joint fluid or histopathology evaluation of synovial biopsy.

Herein we report a case of tuberculous mono-arthritis affecting the right knee joint in a patient with rheumatoid arthritis (RA). We think this is a diagnostic challenge encountered in clinical rheumatology. This is also a very rare presentation in the literature where cases from Sri Lanka have not been reported previously even though Sri Lanka is an endemic country for tuberculosis.

## 2. Case History

A 42-year-old Sri Lankan-Sinhalese male presented with right knee joint swelling and pain for one year. Knee joint pain was associated with features of inflammatory arthritis in other small and large joints of the limbs. He experienced intermittent fever and progressive loss of weight for the same duration.

He was a patient with long-standing history of rheumatoid arthritis (RA) and interstitial lung disease. At the very first presentation to the rheumatology clinic, 26 peripheral joints were involved including all proximal interphalangeal (PIP) joints, metacarpophalangeal (MCP) joints, and both wrist joints. His joint symptoms persisted for 3-month duration. Though both rheumatoid factor (RF) and anti-cyclic citrullinated peptide (anti-CCP) were negative, initial erythrocyte sedimentation rate (ESR) and C-reactive protein (CRP) were elevated. Diagnosis of RA was initially made based on 2010-EULAR/ACR criteria in which his score was 7.

Current medications include methotrexate (MTX) 7.5 mg weekly, sulfasalazine (SSZ) 1 g thrice a day, leflunomide (LEF) 10 mg daily, and mycophenolate mofetil (MMF) 1 g twice a day. Despite good drug adherence, control of disease activity is poor during last two years with high disease activity score (DAS28 > 5). Hence, to achieve low disease activity, we planned for rituximab therapy which was not readily available in our resource-poor setting. Oral prednisolone has been prescribed intermittently to relieve his symptoms.

**Figure 1:**
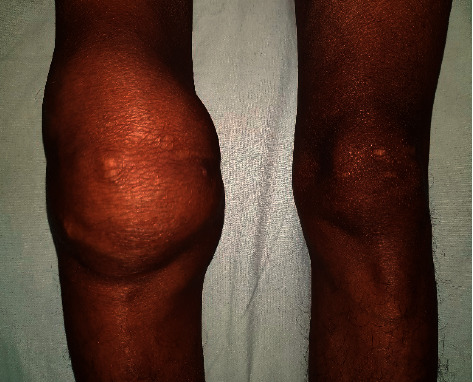
Swollen right knee joint on clinical examination.

Examination revealed swollen and tender right knee joint with effusion and limited range of motion (Figure 1). Proximal interphalangeal, metacarpophalangeal, wrist, elbow, and shoulder joints were symmetrically involved with tenderness. Rest of the examination was unremarkable.

**Figure 2:**
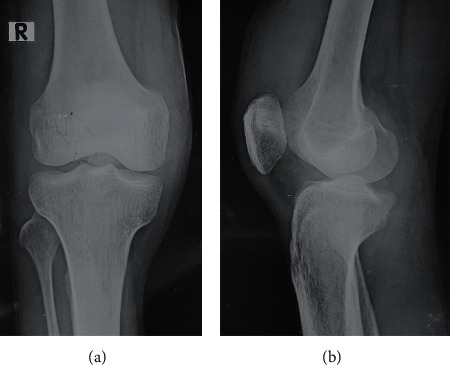
Plain radiograph of right knee joint: anteroposterior view (a); lateral view (b).

His ESR was persistently elevated above 50 mm/hr that was attributed to active synovitis. Other investigations were as follows: white blood cell count, 6600/mm^3^ with 72% neutrophils; platelet count, 480 000/mm^3^; hemoglobin, 12.0 g/dL; CRP, 14 mg/L; and serum uric acid, 6 mg/dL. Plain radiographs of right knee joint were normal except for soft tissue swelling (Figure 2). Ultrasonography of the knee showed joint effusion and marked synovial thickening.

During last one year, his right knee joint has been aspirated several times due to recurrent accumulation of joint fluids causing significant discomfort. The most recent (one month ago) findings of joint fluid analysis were as follows: polymorphs, 18240/mm^3^; lymphocytes, 60320/mm^3^; and red cells, nil/mm^3^. Routine bacteriology culture did not yield any organism in multiple occasions. Uric acid crystals or malignant cells were never identified on those samples. Before eight months of this presentation, the patient was treated with empirical intravenous antibiotics even for the suspected septic arthritis. Intra-articular steroid was injected to the right knee joint on two occasions afterwards.

During a routine clinic visit, we reconsidered the possibilities for resistant knee joint effusion and sent the joint fluid for acid fast bacilli (AFB) and polymerase chain reaction for TB (TB-PCR), and both came positive. He was immediately started on anti-tuberculous therapy upon diagnosis.

His respiratory problem was reevaluated with computed tomography (CT) of chest that further confirmed non-specific interstitial pneumonia (NSIP). The Mantoux test was non-reactive. AFB and TB-PCR on broncho-alveolar lavage (BAL) were negative. He was subjected to HIV screening test that also came negative.

## 3. Discussion

We confirmed the diagnosis of TB of knee joint with positive AFB on microscopic evaluation and positive TB-PCR of joint fluid aspiration. Synovial biopsy was not performed. TB was not contemplated for the right knee joint swelling with effusion on initial presentation because RA was well established, and the patient had high disease activity involving other large joints of the limbs, and the affected knee did not show features of joint destruction despite marked soft tissue swelling on the imaging. Possibility of septic arthritis and secondary osteoarthritis was considered which delayed the investigation for TB.

Our patient was on multiple immunosuppressive disease modifying anti-rheumatic drugs (DMARDs), namely, MTX, SSZ, LEF, MMF, and prednisolone, to control his disease. Association between use of DMARDs and TB is well established in literature [5, 6]. A Chinese study which analyzed 42 patients of RA complicated with TB found that most common site of involvement of these patients was lung (31 cases representing 73.3%); only 6 cases (14.3%) had TB of joints [7].

Though TB is increasingly reported along with HIV/AIDS, primary bone infection remains uncommon [8]. Patients with chronic illness, particularly autoimmune rheumatic diseases, are more vulnerable to acquire skeletal TB owing to their damaged joints following immunological dysfunction and disease modifying immunosuppressive medications. Nevertheless, tuberculous arthritis of knee joint in patients with RA was seldom reported in the literature [9, 10]; two patients presented in these case reports were on biological DMARDs: tumor necrosis factor-alpha (TNF-*α*).

Diagnosis of TB of knee joint remains challenging owing to its rarity, non-specific symptoms, atypical indolent clinical course, low sensitivity, and low specificity of available diagnostic tools, particularly when associated with other common joint disorders such as RA. Triplett et al. reviewed thirteen cases of TB arthritis of knee joint with delayed diagnosis [11]. Five of them were patients with autoimmune rheumatological disorders such as RA and Sjogren's syndrome. In this case series, delaying of diagnosis ranged from two months to ten years. Delay is significantly longer in developed nations where TB is uncommon [4].

## 4. Conclusion

TB should be considered as an important differential diagnosis for chronic mono-arthritis of knee joint with a high degree of suspicion. Particularly where TB is endemic, clinicians should reconsider the possibility of tuberculous septic arthritis, even when well-established another background diagnosis is present to explain the joint symptoms.
